# Effect of the Addition of Soybean Protein and Insect Flours on the Quality of Cooked Sausages

**DOI:** 10.3390/foods13142194

**Published:** 2024-07-11

**Authors:** Nikolay Kolev, Desislava Vlahova-Vangelova, Desislav Balev, Stefan Dragoev, Krasimir Dimov, Evgeni Petkov, Teodora Popova

**Affiliations:** 1 Department of Meat and Fish Technology, University of Food Technologies, 26 Maritsa Blvd, 4002 Plovdiv, Bulgaria; d_vangelova@uft-plovdiv.bg (D.V.-V.); d_balev@uft-plovdiv.bg (D.B.); s_dragoev@uft-plovdiv.bg (S.D.); 2 Agricultural Academy, Institute of Cryobiology and Food Technologies, 53 Cherni vrah Blvd, 1407 Sofia, Bulgaria; krasimir.dimov@ikht.bg; 3 Agricultural Academy, Institute of Animal Science-Kostinbrod, Pochivka St, 2232 Kostinbrod, Bulgaria; e_petkov@ias.bg

**Keywords:** insect flours, soybean protein, sausages, technological characteristics, lipid oxidation, sensory quality, storage

## Abstract

This study aimed to assess the effect of the addition (2%) of soybean protein (SP) and insect flours derived from house crickets (*Acheta domesticus*, HCF) and yellow mealworm (*Tenebrio molitor*, YMF) in cooked sausages. The technological characteristics of the batter, the chemical composition of the sausages, their technological traits and lipid stability during refrigerated storage, as well as their sensory properties, were investigated. The SP, HCF and YMF batters displayed higher pH (*p* = 0.0025) and stability (*p* < 0.0001) but a darker colour (*p* < 0.0001) than the control samples. The addition of SP increased the plasticity of the batter (*p* = 0.0017), while YMF decreased its structural strength (*p* = 0.0274). Higher pH and darker colour were detected in SP-, HCF- and YMF-containing sausages; however, the effect of the alternative proteins depended on the duration of storage. The plasticity decreased in the insect-containing sausages (*p* = 0.0010) and increased over time (*p* = 0.0136), whereas the elasticity was lower in the YMF group (*p* < 0.0001). The protein and fat contents were higher (*p* < 0.0001) in the sausages containing alternative protein. TBARS content decreased over time in these groups. The HCF and YMF sausages received lower scores for their appearance, colour, texture, flavour and taste, suggesting the need for further technological interventions to make such products more attractive to consumers.

## 1. Introduction

One of the most significant challenges in the current food system is feeding the increasing population and achieving food security. According to the UN, from an estimated 2.5 billion people in 1950, the global population reached 8.0 billion people at the end of 2022 and is expected to increase by nearly 2 billion people in the next 30 years [1]. As highlighted by the FAO, this overpopulation of the planet can result in future food insecurity, which has already driven the increase in global protein demand seen over the past decade. In order to meet the increased requirements, the meat supply worldwide should double by 2030.

The growing world socio-economic crisis seen after the COVID-19 pandemic has drawn attention to the importance of nutritious and healthy food for maintaining a strong immune system. The shortage of wholesome food, the pollution of the planet during its production and the rising prices of food products against the background of an energy, social and environmental crisis prompt governments, scientists and ecologists to look for potential food sources to solve these problems. Hence, there is a growing search for alternative food sources such as fungi, cultured meat, micro- and macroalgae and insects. For centuries, the latter have been used as food. A study by Omuse et al. [2] identified 2205 insect species consumed in 128 countries, with Asia having the highest number at 932 species, followed by North America (mainly Mexico (450 species)) and Africa. The use of insects as a component of the diet is more widespread in poorer countries with a significant shortage of wholesome food. Although insect consumption in Europe has been historically well documented since ancient times [3,4], yet the practice remains unpopular in Western societies nowadays. In Europe, insects make up 2% of food, while in America, consumption reaches 39% and in Africa, they comprise 30% of total food consumption [3]. The increasing interest in insect-based products, triggered by the environmental, economic and food security benefits that insects could offer, has led to record growth in the mass production of edible insects. Their demand as a future source of protein is projected to reach 3 million tons by 2030 [5]. The criteria used for the selection of insects for food or feed are based on the possibilities for automation, the use of cheap substrates, market potential and disease avoidance [6]. The yellow mealworm (*Tenebrio molitor*) and crickets (*Acheta domesticus*) are sustainable novel foods with a favourable amino acid profile that offer a suitable alternative to animal protein [7].

Meanwhile, the growing interest in high protein diets and protein-fortified food has caused flooding of the market in recent years. High-protein diets are often associated with a healthy lifestyle; however, the resulting excessive meat consumption is criticized [8].

Based on Commission Implementing Regulation (EU) 2022/169 of 8 February 2022, which authorised the placing on the market of frozen, dried and powdered forms of yellow mealworm (*Tenebrio molitor* larva) as a novel food under Regulation (EU) 2015/2283 of the European Parliament and of the Council, and by amending Commission Implementing Regulation (EU) 2017/2470, the consumption of yellow mealworm was finally allowed. Furthermore, as an amendment of Commission Implementing Regulation (EU) 2017/2470, Commission Implementing Regulation 2023/5 authorised the placing on the market of *Acheta domesticus* as a partially defatted powder, for use as a novel food.

Sausages with added flour from yellow mealworms and house crickets have potential prospects for development. They can offer some advantages and innovations in the food industry, such as a high protein content, diverse taste and texture, sustainability of production and marketing potential [9,10]. The growing interest in alternative protein sources and sustainable foods may contribute to the successful introduction of these products to the market [11]. However, the development of meat products containing insect protein warrants additional research. A previous study evaluated the addition of cricket powder from *Acheta domesticus* to cooked poultry meatballs. The authors concluded that a 2% addition of cricket powder was the most suitable percentage for cooked meat products [12]. Soybeans, among other vegetables, are often used as supplements or substitutes in meat industry. This is due to their high-quality protein, with a well-balanced amino acid profile and functional properties that include emulsification and fat- and water-binding qualities, which improve the technological parameters of meat products [13,14,15]. These qualities make soybeans an alternative source of protein that is also more economically viable. In addition, recent clinical studies demonstrated the health benefits of soybeans on chronic diseases and their preventive effects against some cancers [13]. Driven by the rising interest in alternative protein sources applied in the food industry, in this study, we aimed to investigate and compare the effect of added soybean protein and flours derived from yellow mealworms and house crickets on the quality of cooked sausages.

## 2. Materials and Methods

### 2.1. Raw Materials

The sausages were manufactured using chilled lean and semi-fat pork. The meat, nitrate salt (0.55% NaNO_2_), sodium tripolyphosphate (STPP) (E451) and white granulated sugar were purchased from the local store. The granular soybean product was supplied by Bobal Boyadzhiev Ltd., Sofia, Bulgaria. The house-cricket flour was supplied by Etno Synergy, Bulgarovo, Bulgaria. The yellow mealworm flour was prepared in the Department of Meat and Fish Technology and the Department of Canning and Refrigeration Technology at the University of Food Technology, Plovdiv, Bulgaria. The proximate composition of the soybean protein, mealworm and cricket flours is presented in Table 1.

### 2.2. Formulation and Manufacturing of the Cooked Sausages

Four formulations of cooked sausages were prepared using 6 kg pork (3 kg lean meat from the leg and 3 kg semi-fat meat from the belly), as described in Table 2. The control batter was prepared with lean pork, semi-fat pork, ice water, salt and phosphate. To make the sausages containing alternative protein, soybean granular, mealworm and house-cricket flours were added in the amount of 34.5 g (2%), hydrated in 1/3 of the water at the top of the control recipe. The meat was left to cool (−5 °C) and was ground with a wolf machine to pass through a 5 mm sieve. It was then placed in a cutting machine with the addition of 2/3 of the ice water, nitrite salt and the phosphates and was then emulsified. The batter was separated into 4 equal portions, which were used to prepare the formulations of the cooked sausages. One of the portions served as a control, while for the other three portions, the respective alternative protein sources were added during additional cutting with the rest of the water. After this, the cutting of the batters continued until their temperature reached 14 °C. The batters thus obtained were stuffed into polyamide casings and formed into sausages that were hung on racks. The racks were placed in a scalding chamber, where the sausages were cooked (t = 80–85 °C until reaching 72 °C at the centre). The sausages were then cooled under showers until reaching 20–22 °C in the centre of the product. Fifteen sausages (approx. 400 g) were manufactured from each formulation. The sausages were kept in a refrigerator (0–4 °C) for 7 days.

### 2.3. Laboratory Analyses

A part of each batter was subjected to analysis up to 1 h after preparation. The prepared sausages were then placed in a refrigerator (0–4 °C) without packaging and analysed after 1 and 7 days of storage.

#### 2.3.1. pH and Colour Measurements of the Raw Batter and Cooked Sausages

The values of pH of the batters and cooked sausages were measured with a portable pH meter from Hanna, HI99163 (Hanna Instruments, Inc., Smithfield, RI, USA), equipped with a spear-tipped electrode. Preliminary calibration using standards at pH 4.04 and 6.86 was performed [17]. The colour measurement of each batter and the cross-sections of the cooked sausages were performed using a Konica Minolta CR-410 (Konica Minolta Holding. Inc., Ewing, NJ, USA). The colour coordinates are presented according to lightness, L*, redness, a* and yellowness, b*. The redness index a*/b* was adopted to assess the colour [18]. The colour difference ΔE was calculated according to the equation below:

(1) $$\Delta E=\sqrt{{∆L*}^{2}+{∆a*}^{2}+{∆b*}^{2}}$$

where ΔL*, Δa* and Δb* are the differences between the control and each of the experimental samples.

Both colour and pH measurements were taken from three separate locations of the samples and the results were averaged.

#### 2.3.2. Emulsion Stability

The emulsion stability was determined as described by Álvarez et al. [19], with slight modifications. Immediately after preparation, 40 g of the batter was placed in a centrifuge tube (h = 106 mm, d = 38 mm, h of the sample = ± 50 mm). The tubes were put in a water bath at 72 °C for 30 min. After that, they were centrifuged at 5200 rpm (4226× *g*) for 90 s. The supernatant was separated and weighed. The tubes were then dried at 103 °C for 1 night and were weighed again to estimate the fat exudates. The total expressible fluid (TEF) was calculated as follows:

(2) $$\mathrm{TEF}\%=\frac{Ws-Wp}{Ws}\times 100$$

where Ws—weight of the sample before centrifugation and Wp—weight of the pellet.

(3) $$Water\;form\;TEF,\%=\frac{m_{1}-m_{2}}{m_{1}}\times 100$$

(4) Fat form TEF,%=100−Water from TEF

Here, m_1_—initial mass of the supernatant and m_2_—mass of the dried supernatant.

#### 2.3.3. Texture Analysis

The texture analysis was performed using an OB-5 penetrometer (Labor, Hungary) as described by Bourne [20], with slight modification by Vassilev et al. [21]. The batters were placed in a metal dish (20/20/30 mm). Both batter and cooked sausages were tempered for 30 min at room temperature prior to analysis. Cubes sized 1/1/1 cm were cut from each sausage. Nine replicates for each sample were measured. The structural strength involved compression for 10 s using a round tip attached to the penetrometer holder. The depth was reported on the scale and the structural strength was calculated according to the following equation:

(5) $$Structural\;strength=\frac{K*F}{h^{2}},g/{\mathrm{cm}}^{2}$$

where:

K—constant of the tip (k = 1.853)
F—weight of the holder system, g (107.8)
h—depth, cm

Plasticity. The measurement was carried out as described above using a sharp tip attached to the holder. The plasticity was calculated using the equation below:

(6) $$Plasticity=\frac{K*F}{h^{2}},g/{\mathrm{cm}}^{2}$$

where:

K—constant of the tip (k = 1.853)
F—weight of the holder system, g (103.8)
h—depth, cm

Elasticity. This trait shows the capacity of the product to restore its initial size and shape after removing the deforming pressure. It was calculated as follows:

(7) $$Elasticity=\frac{h-h_{1}}{h},\%$$

where:

h—recorded depth, cm
h_1_—depth recorded after 60 s of rest, cm.

#### 2.3.4. Proximate Composition of the Cooked Sausages

The total nitrogen value was determined using the AOAC [22], and the protein content was calculated using a nitrogen to protein conversion factor of 6.25. The fat content was determined through extraction with a Soxhlet apparatus [23]. The total ash value was measured after incineration at 400–600 °C [24]. The moisture content was calculated after drying at 104–105 °C using a KERN MLS 653A moisture analyser (Kern & Sohn Gmbh, Ballingen, Germany) until it reached a constant weight [25]. The carbohydrate content was calculated using the following equation:

Carbohydrates = 100 − Protein − Fat − Ash − Moisture, %.(8)

#### 2.3.5. Acid Value

The lipid hydrolysis value was described using the acid value of the extracted lipids and measured as described by Kardash and Tur’yan [26]. The extraction of lipids was performed according to the method used by Bligh and Dyer [27]. After extraction, 1 g of lipid was dissolved in 20 mL neutral alcohol-ether mixture (1:1, *v*/*v*), with added phenolphthalein. The mixture was titrated with 0.1 N KOH and the volume of the KOH used was recorded. The acid value was calculated as AV = (V × F × 5.6104)/m, mg KOH/g; where:

V—volume of KOH used for titration, g
F—factor of 0.1 NKOH, 0.996
m—the weight of the sample, g.

#### 2.3.6. Peroxide Value (PV)

The peroxide value was determined as described by Shantha and Decker [28]. The extracted lipid (0.1 g) was mixed in a glass tube with 50 μL iron (II) solution, 50 μL NH_4_ SCN (300 mg/mL) and CHCl_3_:CH_3_OH (3:5, *v*/*v*), to reach a final volume of 10 mL. The samples were incubated for 5 min at room temperature and the absorbance was determined by spectrophotometer at 507 nm against a blank containing all the reagents except the sample. The results were presented as meqO_2_ /kg lipid.

#### 2.3.7. TBARS Content

The content of the thiobarbituric acid reactive substances was determined as described by Botsoglou et al. [29], with slight modifications by Kolev et al. [30]. Briefly, 10 g of the sample was homogenized with 50 mL NaCl (0.9%) and left for 5 min. Then, 50 mL of trichloroacetic acid (10%) was added and the samples were filtered through Filtrax (grade 391). The filtrates (4 mL) were then mixed with 1 mL 2-thiobarbituric acid (1%) and were incubated at 70 °C for 30 min. After cooling, the absorbance of the samples was determined at 532 nm against a blank containing distilled water. TBARS concentrations were calculated using 1, 1, 3, 3-Tetraethoxypropane as standard. The results were expressed as mg MDA/kg product or TBARS units.

#### 2.3.8. Sensory Analysis

The sensory analysis was performed according to a 5-point hedonic scale, where: 1—dislike very much; 2—dislike slightly; 3—neither like nor dislike; 4—like slightly; 5—like very much [31]. The analysis was performed by five trained panellists whose reactions were tested through a triangle test [32]. The sensory traits thus assessed were appearance, colour of the cutting surface, taste, flavour and texture.

#### 2.3.9. Statistical Procedures

The statistical evaluation was performed using the JMP software v.7 [33]. The effect of the alternative protein sources on the traits of the batter and the chemical composition of the cooked sausages was evaluated through a one-way ANOVA. The effect of the protein type, the duration of storage and their interaction was assessed through a two-way ANOVA. Whenever needed, the differences in means were determined through post hoc comparisons (Tukey HSD, *p* < 0.05).

## 3. Results

### 3.1. Technological Characteristics of the Batter

#### 3.1.1. pH and Instrumental Colour of the Batter

The addition of alternative protein to the batter was associated with a significant increase in the pH (*p* < 0.0025). It varied within the range of 5.90–5.93 when compared to the control batter (pH = 5.80).

As seen in Table 3, there was a significant effect of the alternative protein source on the instrumental colour parameters of the batter (*p* < 0.0001), showing lower values of L* and a* and higher values of b* in the treated groups, when compared to the control. The brightest colour was observed in the HCF batter. The addition of cricket flour also led to lower redness and higher yellowness in comparison to the SP and YMF groups. The latter showed lower values of L; however, the values of a* and b* differed only slightly. The values of the a/b ratio differed significantly among the groups (*p* < 0.0001), these values being lower in the samples containing the alternative protein source in comparison to the control. Furthermore, the HCF group displayed the lowest a*/b* ratio. The protein source also affected the values of ΔE. Among all the treated groups, the YMF batter displayed higher values of this parameter, corresponding with the lower L* value measured in this group.

#### 3.1.2. Stability and Texture Profile of the Batter

The stability of the emulsion in terms of total expressible fluid showed that regardless of the protein source, the SP, HCF and YMF batters had higher stability and all of them had a lower percentage of TEF in comparison to the control batter (Table 4). In addition, the lowest TEF percentage was observed in the HCF group, followed by the YMF and SP groups. At the same time, a dependence was observed between the type of added alternative protein source and the percentage ratio of water/fat from TEF. The batter with added insect flours (HCF and YMF) was characterized by a higher percentage of fat from TEF compared to the control C and SP batters. The type of protein source also affected the texture of the batter. Both the HCF and YMF groups displayed considerably lower plasticity when compared to the SP batter. With regard to the structural strength, the lowest value of this parameter was observed in the YMF batters, while the HCF group remained in an intermediate position.

### 3.2. Proximate Composition of the Cooked Sausages

The addition of the alternative protein source affected the content of the proteins and fat in the sausages (*p* < 0.0001) and was associated with an increase in these parameters, compared to the control (Table 5). Both groups of sausages containing insect protein displayed higher contents of protein and fat in comparison to the SP group. The higher contents of protein and fat in the HCF and YMF groups corresponded with the lower moisture content in these sausages than in both the control and SP groups (*p* = 0.0039). The ash content was also considerably increased in the sausages containing both soybean and insect proteins (*p* < 0.0001), with the YMF group showing higher values of this indicator. The carbohydrate content did not display any significant differences among the groups; however, it tended to be higher in the YMF group of sausages.

### 3.3. Technological Parameters of the Cooked Sausages

#### 3.3.1. pH and Instrumental Colour

The pH values of the sausages depended on both the protein source and storage period (*p* < 0.0001) (Table 6). The addition of insect protein led to a significant increase in the pH of the HCF and YMF groups, while the control and SP groups had similar values. The storage period was also associated with an increase in pH; however, this was not observed in the YMF group.

As seen in Table 6, there was a significant interaction between treatment and storage period with regard to all the colour indicators of the cooked sausages. The addition of insect protein was associated with a significant decrease in L* and a* in the HCF and YMF sausages when compared to the C and SP groups. Conversely, regardless of the alternative protein source, L* decreased between 1 and 7 days of storage in all the groups, including the control. No such trend was observed regarding the a* values. The latter considerably decreased with time in only the HCF group. In comparison to the control sausages, b* increased in the SF and YMF groups. Except in the HCF-containing sausages, in all the rest, there was a significant increase in yellowness during the refrigerated storage period. The values of the a/b ratio were lower in the SP, HCF and YMF groups and, over time, they decreased in all the groups except the SP group, which corresponded to the slight changes observed in the a* and b* values in this group during storage. A greater colour difference was observed in the HCF and YMF groups compared to the SP group. Conversely, the values of this trait showed contradictory patterns with regard to the storage period. While in the SP and YMF groups, this indicator tended to decrease, in the HCF group, there was a considerable increase in ΔE during the storage period.

#### 3.3.2. Texture Profile

The plasticity of the sausages was affected by both the presence of alternative protein (*p* = 0.001) and the storage period (*p* = 0.0136) (Table 7). The SP groups displayed higher plasticity when compared with all the other groups, whereas this parameter decreased considerably in the HCF and YMF groups. For all the groups, the plasticity increased during storage. This was also observed with regard to the structural strength (*p* = 0.0419). The elasticity was only affected by the additional protein source (*p* < 0.0001), showing considerably lower values in the YMF group.

#### 3.3.3. Changes in Lipid Stability

The alternative protein source and the storage time exerted different effects on the lipids of the sausages (Table 8). The hydrolysis in the lipid fraction was significantly affected by both factors. Regardless of the protein source, AV increased in all the experimental groups (*p* < 0.0001), with considerably higher values observed in the HCF and YMF groups. In all the groups, AV increased significantly over time (*p* = 0.0001). The content of the primary lipid oxidation products, as expressed by PV, showed negligible differences among the groups; however, it decreased during the storage period. With regard to TBARS, both factors significantly interacted (*p* = 0.0309). The SP and YMF groups displayed significantly lower TBARS contents in comparison to the C and HCF groups. While the control group remained stable during the storage period and the YMF group showed insignificant changes, in the SP and HCF groups, the TBARS values decreased during storage.

#### 3.3.4. Sensory Evaluation

**Figure 1:**
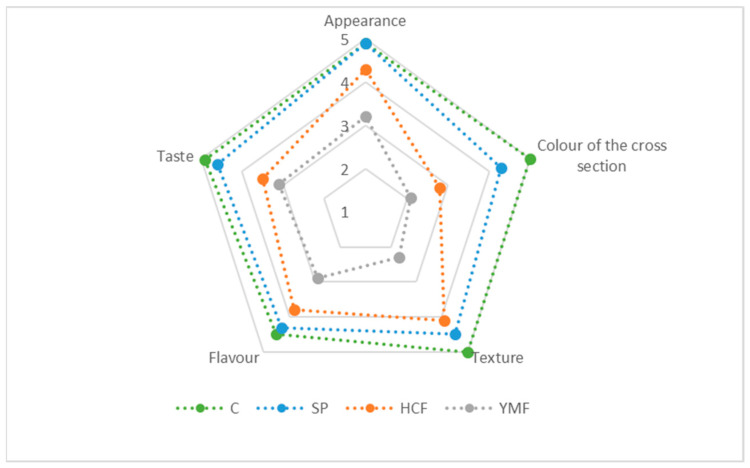
Sensory evaluation of the sausages containing soybean protein, house-cricket flour or yellow mealworm flour.

The addition of insect protein negatively affected the sensory evaluation of the sausages. As presented in Figure 1, both the HCF and YMF groups received lower scores from the panellists for all the sensory traits. Furthermore, the sausages containing yellow mealworm flour received lower scores regarding appearance, texture and flavour.

## 4. Discussion

The values for pH that we measured in this study showed a significant increase in the groups containing insect flour before cooking. This was not surprising and can be attributed to the high pH of the yellow mealworm flour [16] and house-cricket flour [34]. Similar results were reported by Kim et al. [35] after adding flours made from *Tenebrio molitor*, *Allomyrina dichotoma* and *Protaetia brevitarsis seulensis* to meat emulsions. Additives increasing the pH of batter also increase its water-holding capacity. This is a positive trait in the supplements used in manufacturing cooked sausages. The higher pH of the batter in the groups containing soybean or insect flours corresponds to higher emulsion stability, as expressed by the TEF. A close relationship between the proximate composition and the composition of the TEF was observed. With the decrease in moisture and increase in fat content, the percentage of water from the TEF decreased while the percentage of fat from the TEF increased. A higher pH in the HCF and YMF groups was also observed after cooking. In line with this finding, Vlahova-Vangelova et al. [34] showed an increased pH in cooked sausages containing house-cricket flour. Such results were also reported by Choi et al. [36]. Conversely, Kim et al. [37] did not observe any effect on pH after the replacement of the meat with 5 and 10% cricket flour regarding both batter and cooked sausages. With regard to the soybean protein, its addition to the batter led to a higher pH; however, after cooking, the pH of the SP sausages did not differ from the control samples. This contradicts the results of Kang et al. [14], who reported a significant increase in pH in the batter of chicken Vienna sausages containing soybean, a value that also remained after cooking.

The higher pH in the batter of the SP, HCF and YMF groups corresponded to a considerable decrease in lightness and redness. Furthermore, this was also observed after cooking, both in the prepared sausages and during storage under refrigerated conditions. Such an effect from the addition of soybean protein and insect flours was confirmed in other studies [14,36,38,39]. Considerable discolouration, as demonstrated by the decreasing of the a*/b* ratio, was observed in the batter and sausages containing soybean or insect supplements. The values of this index also decreased during storage in the insect-supplemented sausages. In addition, both the HCF and YMF groups displayed high values of ΔE in the batter and after cooking that correspond to the low a/b ratio. In line with our results, Han et al. [9] observed that the addition of cricket flour led to considerable changes in sausage colour, more notably with 2.5% and 5% supplementation. Zhang et al. [40] also reported a significant colour difference in frankfurters containing the *Tenebrio molitor* larvae protein. According to Lenaerts et al. [24], a colour difference below 1.5 is invisible, while a difference of ΔE* > 6 is clearly visible. In this study, the ΔE* in both the experimental batters and sausages did not exceed 6. However, it should be mentioned that sausages containing SP displayed a considerably lower colour difference than the batter. It might be suggested that the incorporation of soybean protein prevents colour changes after cooking.

The texture profile of the batter revealed low structural strength in the groups containing insect protein, in contrast to the soybean protein-containing samples. Conversely, after cooking, all the groups with protein supplements exhibited lower structural strength and plasticity in comparison to the control samples. This was particularly noticeable in the SP groups, where there was a dramatic decrease in the structural strength of the sausages. Such results are in line with those of Kang et al. [14]. In agreement with our results, Han et al. [9] reported lower levels of hardness and cohesiveness in cooked sausages containing 1–5% cricket flour. The addition of *Tenebrio molitor* (10 and 20%) as a replacement for pork in sausages also led to lower levels of hardness [41]. Similar results were also reported in other studies [36,42]. Lemke et al. [41] hypothesized that the negative effect of the insect flours on the structure of the cooked sausages might be attributed to a decreased solid content due to the lower protein content. However, this was not observed in our study, since a significantly higher content of protein was measured in the HCF and YMF groups when compared to the control. It might be suggested that the decreased values of structural strength and hence, decreased toughness might be due to the higher water-holding capacity of the products. Vlahova-Vangelova et al. [16] also speculated that the mixture of different proteins, such as insect and plant proteins, might not be able to aggregate in the gel structure, thus leading to a decrease in the structural strength. According to Scholliers et al. [43], the structures of batters containing insect larvae (*A. diaperinus*, *T. molitor* and *Z. morio*) were characterised as weak gels with solid-like behaviour. However, the authors demonstrated that the insect larvae have inferior structure-forming capacities in comparison to meat, which depend on the heating temperature. The lower structural strength of the SP, HCF and YMF sausages was relevant to the lower texture score obtained in the sensory evaluation.

The proximate composition of the sausages showed that the samples containing protein additives, particularly in the HCF and YMF groups, have significantly higher protein levels than the control group. In addition to the higher protein content, the sausages containing soybean or insect flours displayed a higher fat content. This is in agreement with the results of Kim et al. [44] when replacing pork with untreated yellow mealworm larvae and silkworm pupae. Choi et al. [36], when replacing 5–30% of pork with yellow mealworm flour in frankfurters, also found that the fat content increased in those sausages containing a lower percentage of insect flours, and considerably decreased with an increase in the insect flour percentage. In contrast to our results, Cruz-López et al. [45] did not find any change in the fat content of sausages made with grasshopper flour, while Ho et al. [11] reported a significant decrease in the fat content of sausages containing domestic cricket flour. The higher protein and fat contents of SP-, HCF- and YMF-containing sausages corresponded to their notably lower moisture levels in comparison to the control samples. This was also observed by Han et al. [9] when adding different percentages of cricket flour in cooked sausages and by Cruz-López et al. [45] in sausages with the addition of grasshopper flour.

An elevated AV was observed in the groups of sausages containing soybean or insect protein, indicating the intensive hydrolysis of the lipids in these groups. This corresponds to the higher lipid content of the SP, HCF and YMF groups. The acid value increased in the course of storage; however, this increase occurred to a lesser extent in the insect-containing sausages. The peroxide values tended to be lower in the HCF and YMF groups on day 1 of storage and decreased considerably in all the experimental groups until day 7. Except for the HCF group on day 1 of storage, the other sausages containing alternative protein exhibited lower TBARS levels in comparison to the control, and their TBARS levels decreased over time. It can be noted that the YMF sausages displayed considerably lower levels of TBARS. Contrary to our results, the incorporation of pretreated mealworm larvae and silkworm pupae led to higher TBARS values in emulsified sausages, as reported by Kim et al. [44]. Our results also contradict those of Han et al. [9]. Conversely, in agreement with our results, Jeon et al. [46] assessed the oxidative stability of emulsified sausages with different levels of *Tenebrio molitor*, observing a lower TBARS content in the insect-containing samples. The authors suggested that this might be due to the antioxidant potential of *Tenebrio molitor*, which was reported by Kim et al. [47]. In a previous study [48], we also demonstrated the strong antioxidant activity seen in yellow mealworm flours, which might explain the low level of secondary oxidation products. The values of the indicators of lipid oxidation in this study remain below the set limits (PV < 2 meq O_2_/kg; TBARS < 2 mg/kg) [49,50], indicating a low degree of oxidation in all the groups, regardless of the storage time.

A key requirement in sausages containing proteins of vegetable or insect origin is that they should have the same taste and texture as ones made entirely of meat. At the same time, the biological value in terms of nutrients should be preserved [51]. According to Schreuders et al. [52], the leading factors that determine the consumers’ choice of hybrid products are their attitude towards alternative proteins and their concerns about or willingness to adopt new foods. It is generally accepted that the ingredients in meat analogues compromise their taste, lowering their organoleptic characteristics. Meat analogues must possess a number of characteristics to satisfy consumer requirements. Such characteristics are a good appearance, aroma, taste and texture. The taste and texture of meat products are highly valued and are crucial in terms of consumer choice. Plant proteins seem to be a suitable alternative to improve the sensory profile of meat products. Texturized plant proteins are often used to enhance the texture and appearance of real meat [53]. Grasso et al. [54] reported a significantly improved overall level of acceptance and flavour for beef meatballs containing 15% textured soy protein and yeast. This is in agreement with our results, which show a high score for overall appearance and texture in the sausages containing soybeans. Due to the accumulation of many ingredients, it is difficult to achieve a homogeneous colour in meat products with the addition of non-meat proteins. This may be due to the fact that meat analogues consist of several ingredients, while meat is an entire muscle group [55]. In contrast to the addition of soybeans, the incorporation of insect flours in meat products is an issue of concern regarding their impact on the products’ sensory properties. Overall, the sensory evaluation of the sausages containing insect protein in this study was associated with lower scores. In line with our results, Cavalheiro et al. [38] reported different sensory evaluations for frankfurters containing cricket flour, depending on the percentage of replacement. The authors observed lower scores for the sensory traits in sausages containing 5 and 7.5% insect flour. With regard to the sensory quality, later, Cavalheiro et al. [39] demonstrated that the cricket flour percentage should not exceed 5% in beef patties, as levels of 7.5 and 10% of cricket flour were associated with a negative impact. Zhang et al. [40] reported the effect of the pre-drying treatment of *Tenebrio molitor* larvae when incorporated in frankfurters and also the effect of the percentage of flour replacing meat. When compared to the control group, the incorporation of flour from freeze-dried larvae (5, 10 and 15%), as well as microwave-dried larvae (10 and 15%), had a negative effect on the sensory characteristics of the meat products. Generally, the incorporation of insect flours in meat products has either a negative or no effect on the sensory profile of the products, especially in profiles associated with colour and taste. The darker colour observed in the batter and sausages, particularly in those containing insect flours, in our study corresponds to the lower sensory evaluation of HCF- and YMF-containing cooked sausages regarding their appearance and colour. Among them, a darker colour of the batter and sausages was recorded for the YMF group. This corresponded to the lowest scores concerning the appearance and colour of the product containing yellow mealworm flour. This suggests the need for additional technological interventions that will enhance the organoleptic properties of hybrid products to make them more attractive to consumers.

## 5. Conclusions

The results of this study demonstrated both the advantages and disadvantages of soybean granular protein, as well as cricket and mealworm flours, when acting as protein supplements in cooked sausages. The effect of the incorporation of these protein sources on the examined traits of the sausages, however, depended on their kind. The inclusion of soybean and insect proteins resulted in a higher pH and stability of the batter; however, they were also associated with a darker colour, lower levels of redness and increased yellowness. The soybean protein increased the plasticity of the batter, while the yellow mealworm protein decreased its structural strength. After cooking, the effect of the alternative proteins remained the same with regard to pH and colour; however, this depended on the time of storage. In contrast to the batter, the structural strength of the sausages was only affected by the duration of storage, whereas the plasticity level was lower in the insect-containing sausages. The elasticity was considerably lower in the sausages containing yellow mealworm flour. Additionally, the alternative protein source that was incorporated in the sausages increased the protein and fat content, while, with regard to lipid oxidation, these groups remained stable. The sausages containing insect flours exhibited markedly lower scores for their overall appearance, colour, texture, flavour and taste, suggesting the need for further technological interventions to make such products more attractive to consumers.

## Figures and Tables

**Table 1:** Proximate composition of the protein ingredients used in manufacturing the cooked sausages.

Item	Soybean Protein (SP) *	House-Cricket Flour (HCF) *	Yellow Mealworm Flour (YMF) **
Moisture, %	8.52	10.47	1.03
Ash, %	5.92	4.10	3.00
Protein, %	60.00	58.10	62.46
Fat, %	10.16	14.43	14.04
Carbohydrates and fibres, %	15.40	12.90	19.47

* Data declared by the producer; ** data analysed in the Laboratory of Meat Technology [16].

**Table 2:** Formulations for the cooked sausages used in the study.

Ingredients	Groups			
	Control (C)	SP	HCF	YMF
Pork (lean), g	3000	3000	3000	3000
Pork (semifat) g	3000	3000	3000	3000
Water/ice, g	900	900	900	900
Salt (0.55% NaNO_2_), g/kg	20	20	20	20
Phosphates, g/kg	2.0	2.0	2.0	2.0
Soybean granular in the cooked sausage, g	-	34.5	-	-
House-cricket flour in the cooked sausage, g	-	-	34.5	-
Yellow mealworm flour in the cooked sausage, g	-	-	-	34.5

**Table 3:** pH and instrumental colour parameters of the batters, as affected by the alternative protein source.

Treatment	pH	L*	a*	b*	a*/b*	ΔE
C	5.80 ^b^	65.07 ^a^	9.13 ^a^	12.48 ^c^	0.73 ^a^	-
SP	5.90 ^a^	61.79 ^c^	7.07 ^b^	13.07 ^b^	0.54 ^b^	3.92 ^b^
HCF	5.93 ^a^	62.33 ^b^	6.92 ^c^	13.39 ^a^	0.52 ^c^	3.63 ^b^
YMF	5.90 ^a^	61.37 ^d^	6.99 ^bc^	12.94 ^b^	0.54 ^b^	4.30 ^a^
SEM	0.03	0.12	0.05	0.06	0.004	0.13
Sig. (*p*)	0.0025	<0.0001	<0.0001	<0.0001	<0.0001	0.0019

Means connected with different superscripts differ significantly (*p* < 0.05).

**Table 4:** Stability and texture of the batter, as affected by the alternative protein source.

Treatment	TEF, %	Water from TEF, %	Fat from TEF, %	Plasticity, g/cm^2^	Structural Strength, g/cm^2^
C	4.00 ^a^	31.98 ^a^	68.02 ^b^	1.01 ^b^	4.58 ^ab^
SP	3.25 ^b^	30.93 ^a^	69.07 ^b^	2.91 ^a^	4.78 ^a^
HCF	0.28 ^d^	25.04 ^b^	74.96 ^a^	1.09 ^b^	3.62 ^ab^
YMF	1.65 ^c^	23.46 ^b^	76.54 ^a^	0.95 ^b^	3.43 ^b^
SEM	0.14	1.24	1.24	0.44	0.51
Sig. (*p*)	<0.0001	<0.0001	<0.0001	0.0017	0.0274

Means connected with different superscripts differ significantly (*p* < 0.05).

**Table 5:** Proximate composition of the cooked sausages, as affected by the addition of an alternative protein source.

Treatment	Moisture, %	Ash, %	Protein, %	Fat, %	Carbohydrate, %
C	68.72 ^a^	0.70 ^d^	16.81 ^c^	12.28 ^d^	1.49
SP	65.16 ^ab^	1.00 ^c^	17.53 ^b^	13.65 ^c^	2.66
HCF	62.74 ^b^	1.30 ^b^	18.99 ^a^	14.48 ^b^	2.49
YMF	60.07 ^b^	2.00 ^a^	18.40 ^a^	15.64 ^a^	3.89
SEM	1.97	0.05	0.23	0.097	2.02
Significance (*p*)	0.0039	<0.0001	<0.0001	<0.0001	0.5690

Means connected with different superscripts differ significantly (*p* < 0.05).

**Table 6:** pH and instrumental colour measurements of the cooked sausages, as affected by the alternative protein source and the storage period.

Treatment	Storage	pH	L*	a*	b*	a*/b*	ΔE
C	1 d	6.10 ^c^	66.64 ^a^	12.83 ^a^	7.17 ^d^	1.79 ^a^	-
	7 d	6.20 ^b^	64.66 ^b^	13.01 ^a^	7.66 ^c^	1.70 ^b^	-
SP	1 d	6.10 ^c^	65.94 ^a^	12.07 ^b^	7.98 ^bc^	1.51 ^c^	1.41 ^c^
	7 d	6.27 ^a^	64.34 ^b^	12.14 ^b^	8.14 ^b^	1.49 ^c^	1.10 ^c^
HCF	1 d	6.20 ^b^	64.52 ^b^	10.92 ^c^	7.18 ^d^	1.52 ^c^	2.86 ^b^
	7 d	6.30 ^a^	61.70 ^cd^	9.95 ^d^	7.20 ^d^	1.38 ^d^	4.31 ^a^
YMF	1 d	6.20 ^b^	62.44 ^c^	10.69 ^c^	8.10 ^b^	1.32 ^d^	4.80 ^a^
	7 d	6.20 ^b^	61.08 ^d^	10.45 ^cd^	8.57 ^a^	1.22 ^e^	4.52 ^a^
Effects							
Treatment	C	6.15	65.65	12.92	7.42	1.74	-
	SP	6.18	65.14	12.11	8.06	1.50	1.25
	HCF	6.25	63.11	10.44	7.19	1.45	3.59
	YMF	6.20	61.76	10.57	8.34	1.27	4.66
Storage	1 d	6.15	64.89	11.63	7.61	1.54	3.02
	7 d	6.24	62.95	11.39	7.89	1.45	3.31
**RMSE**		**0.02**	**0.32**	**0.22**	**0.15**	**0.03**	**0.38**
Sig. (*p*)	Treatment	<0.0001	<0.0001	<0.0001	<0.0001	<0.0001	<0.0001
	Storage	<0.0001	<0.0001	0.0158	0.0003	<0.0001	0.1389
	Treatment × Storage	<0.0001	0.0060	0.0014	0.0406	0.0263	0.0024

Means connected with different superscripts differ significantly (*p* < 0.05).

**Table 7:** Texture of the sausages, as affected by the alternative protein source and storage period.

Treatment	Storage	Plasticity, g/cm^2^	Structural Strength, g/cm^2^	Elasticity, %
C	1 d	40.48	362.77	1.00
	7 d	42.15	512.33	1.00
SP	1 d	35.62	164.23	1.00
	7 d	84.19	506.26	1.00
HCF	1 d	19.98	195.39	1.00
	7 d	40.14	587.18	1.00
YMF	1 d	9.73	109.55	0.63
	7 d	13.73	176.63	0.65
Effects				
Treatment	C	41.31 ^ab^	437.55	1.00 ^a^
	SP	59.91 ^a^	335.24	1.00 ^a^
	HCF	30.06 ^bc^	391.29	1.00 ^a^
	YMF	11.73 ^c^	143.09	0.64 ^b^
Storage	1 d	26.45 ^b^	207.99 ^b^	0.90
	7 d	45.05 ^a^	445.60 ^a^	0.91
**RMSE**		**16.42**	**263.13**	**0.06**
Sig. (*p*)	Treatment	0.0010	0.2652	< 0.0001
	Storage	0.0136	0.0419	0.8399
	Treatment × Storage	0.0885	0.6769	0.9880

Means connected with different superscripts differ significantly (*p* < 0.05).

**Table 8:** AV, PV and TBARS, as affected by the alternative protein source and storage.

Treatment	Storage	AV, mg KOH/g	PV, meqO_2_ /kg	TBARS, mg MDA/kg
C	1 d	0.47	1.66	0.83 ^ab^
	7 d	0.66	1.41	0.83 ^ab^
SP	1 d	0.59	1.67	0.75 ^c^
	7 d	0.87	1.18	0.67 ^d^
HCF	1 d	0.84	1.61	0.86 ^a^
	7 d	0.89	1.31	0.78 ^bc^
YMF	1 d	0.85	1.59	0.51 ^e^
	7 d	0.97	1.25	0.49 ^e^
Effect				
Treatment	C	0.56 ^c^	1.54	0.83
	SP	0.73 ^b^	1.42	0.71
	HCF	0.87 ^a^	1.46	0.82
	YMF	0.91 ^a^	1.42	0.50
Storage	1 d	0.69 ^b^	1.63 ^a^	0.74
	7 d	0.85 ^a^	1.29 ^b^	0.69
**RMSE**		**0.08**	**0.08**	**0.03**
Sig. (*p*)	Treatment	< 0.0001	0.0780	< 0.0001
	Storage	0.0001	< 0.0001	0.0010
	Treatment × Storage	0.1093	0.0969	0.0309

Means connected with different superscripts differ significantly (*p* < 0.05).

## Data Availability

The original contributions presented in the study are included in the article, further inquiries can be directed to the corresponding authors.
